# A Rare Case of Lethal Neonatal Rigidity and Multi-Focal Seizure Syndrome

**DOI:** 10.7759/cureus.13600

**Published:** 2021-02-27

**Authors:** Palanikumar Balasundaram, Melanie Fijas, Suhas Nafday

**Affiliations:** 1 Pediatrics- Neonatal/Perinatal Medicine, Children's Hospital at Montefiore, New York, USA; 2 Pediatrics- Neonatal/Perinatal medicine, Children's Hospital at Montefiore, New York, USA

**Keywords:** rmfsl, epileptic encephalopathy, brat1 gene, neonatal rigidity, multi-focal seizure, refractory seizures

## Abstract

We present a case of lethal neonatal rigidity and multifocal seizure syndrome (RMFSL) in an early-term female infant born to non-consanguineous parents. RMFSL is a recently discovered autosomal recessive disease caused by the BRAT1 gene mutations. The BRAT1 gene encodes the BRCA1-associated protein required for ATM activation-1, a protein that interacts with BRCA1 and ATM to initiate DNA repair in response to DNA damage. The exon sequence revealed biallelic deletions of exon 1-2 of the BRAT1 gene in our patient. There are only a few cases of RMFSL reported in the literature, and all of them have died before two years, mostly in the first six months of life. Our patient died at the age of 74 days.

## Introduction

Lethal neonatal rigidity and multifocal seizure syndrome (RMFSL) is a severe epileptic encephalopathy that manifests with rigidity and refractory seizures. RMFSL is a recently discovered autosomal recessive disease caused by the BRAT1 gene mutations. The BRAT1 gene encodes the BRCA1-associated protein required for ATM activation-1, a protein that interacts with BRCA1 and ATM to initiate DNA repair in response to DNA damage. Only a few cases of RMFSL have been reported in the literature, and all of them have died before two years, a majority in the first six months of life [1]. The first cases of RMFSL were reported by Puffenberger et al. in 2012, who found a homozygous variant in the BRAT1 gene in two of the three patients [2]. In this case report, we present a case of a homozygous truncating mutation in the BRAT1 gene in a female infant, leading to lethal neonatal rigidity and multifocal seizure syndrome.

## Case presentation

A three-week-old neonate was transferred to our neonatal intensive care unit (NICU) for intractable seizures since birth. She was 37-week gestational at birth. She was symmetric small for gestation age female infant born to non-consanguineous parents by cesarean section. Birth weight was 1790 grams (<0.01 percentile), head circumference was 28.5 cm (<0.01 percentile), and length was 38.1 cm (<0.01 percentile). Physical examination was significant for microcephaly with small anterior and posterior fontanelle, microphthalmia, hypotelorism, high arched palate, single palmar crease, smooth and long philtrum. The neurological evaluation was prominent for central hypotonia with hypertonia of all the extremities, clenched fists, and ankle clonus. The infant had seizures in the form of tonic-clonic movements in the left arm at the initial presentation, which then progressed to generalized seizures. She also had constant twitching movements of the face and tongue and myoclonic jerks upon touch. There was no family history of seizures. The infant had a one-year-old sibling with no medical problems.

The infant was dependent on non-invasive ventilatory support (NIV) throughout her NICU stay except for two days of intubated ventilation at five weeks of age to facilitate a surgical ‘percutaneous left subclavian vein central line’ placement. She remained on NIV until her demise.

Investigations

Complete sepsis evaluation was done, which was negative for bacterial infections. Herpes simplex virus polymerase chain reaction (PCR) was negative in blood and cerebrospinal fluid (CSF). TORCH (toxoplasmosis, rubella, cytomegalovirus, and herpes simplex virus) screen was negative. The cell count, protein, glucose, quantitative amino acids, lactate, and pyruvate in CSF analysis was within normal limits. Serum lactate, pyruvate, ammonia, plasma quantitative amino acids, carbohydrate-deficient transferrin, biotinidase, and very-long-chain fatty acids were normal. She also had a normal coagulation profile, and her plasma acylcarnitine and carnitine profiles were normal. CSF glycine and serine were normal, which ruled out nonketotic hyperglycinemia and serine biosynthesis defects. Vitamin B12 levels were normal. Blood was sent for 'infantile epilepsy panel' gene sequencing.

**Figure 1:**
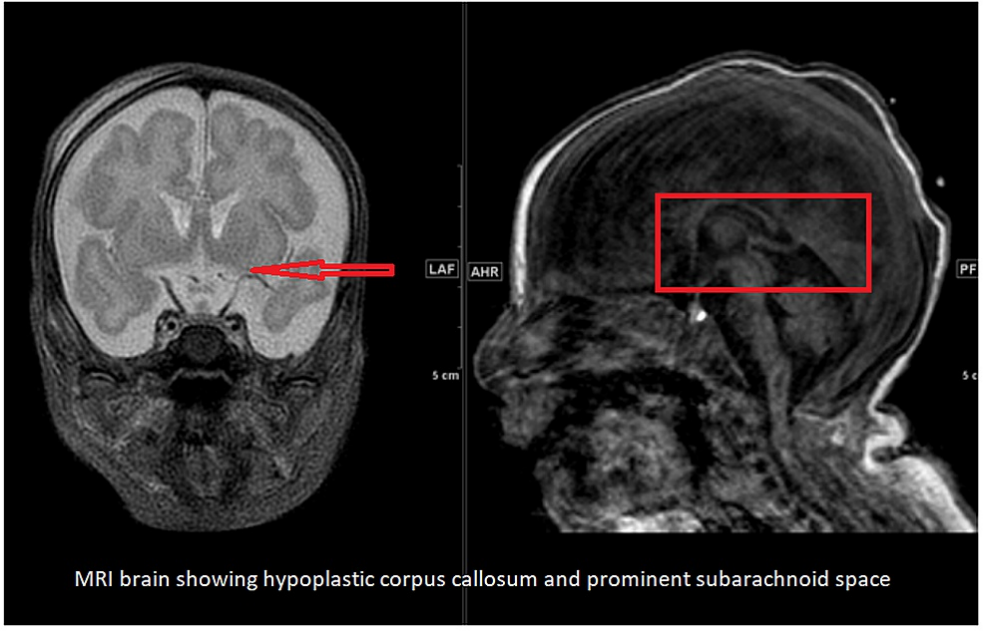
MRI Brain

**Figure 2:**
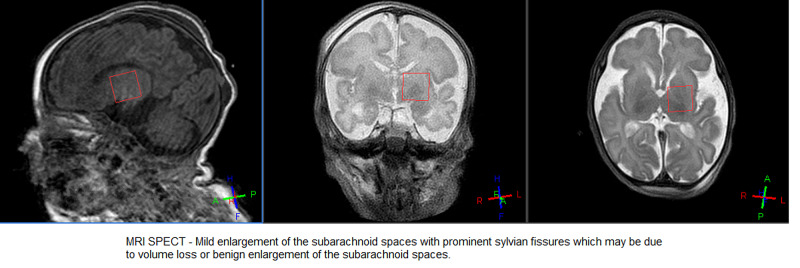
MRI brain spectroscopy

Initial electroencephalogram (EEG) was abnormal, suggestive of moderate to severe epileptic encephalopathy. The interictal record was consistent with severe diffuse cerebral dysfunction. A cranial ultrasound done around one month of age was unremarkable. Brain MRI showed mild enlargement of the subarachnoid spaces with prominent Sylvian fissures, and the corpus callosum was small in size, measuring 1.5 mm in thickness (Figures 1, 2).

Initial genetic testing revealed a microdeletion of the LiNGO1 (Leucine-rich repeat and immunoglobulin domain-containing protein 1) gene. LiNGO1 encodes a protein expressed in the central nervous system. It is part of a large complex that regulates myelination, oligodendrocyte differentiation, axon regeneration, and neuronal survival [3]. Infantile epilepsy panel gene sequencing revealed homozygous deletion involving at least exons 1-2 of the BRAT1 gene, consistent with the diagnosis of autosomal recessive BRAT1 related disorder.

Differential diagnosis

Inherited diseases of metabolism and genetic etiology were also considered in the differential diagnoses. The urine, plasma, and CSF analysis did not reveal any metabolic defects. Complete infantile epilepsy panel gene sequencing was tested to study more than 100 genes associated with refractory seizures, which confirmed lethal neonatal rigidity and multifocal seizure syndrome (RMFSL) due to biallelic BRAT1 mutation.

Treatment

In the NICU, she was treated with high doses of fosphenytoin (8 mg/kg/day), phenobarbital (up to 8 mg/kg/day), levetiracetam (up to 80 mg/kg/day), and later with lacosamide (4 mg/kg/day). A trial of vitamin challenges including pyridoxine (100 mg, one dose followed by 50 mg every day), pyridoxal-5-phosphate (30 mg/kg/day), and folinic acid (10 mg every day) tried from day 27 for two weeks which failed to control seizures. As she did not respond to anti-epileptic drugs and vitamin challenges, the ketogenic enteral diet was started at about six weeks of life. However, the seizures were refractory to the ketogenic diet and four anti-seizure medications. Since the parents agreed to comfort care for the infant, the ketogenic diet was discontinued at the age of 73 days of life.

Outcome and follow-up

The exon sequence revealed biallelic deletions of exon 1-2 of the BRAT1 gene. This lethal condition was discussed with the family in a multi-disciplinary group. After the parent's consent, we decided to redirect care and provide comfort care while withdrawing respiratory support at the age of 74 days. The infant died shortly after with parents at the bedside.

## Discussion

Epileptic encephalopathies have recurrent seizures and interictal epileptiform discharges during the early infantile period [4]. Lethal neonatal rigidity and multifocal seizure syndrome (RMFSL) is a severe epileptic encephalopathy caused by BRAT1 gene mutation. Puffenberger et al. discovered a one-base pair insertion in the BRAT1 gene, resulting in a frameshift and premature termination, and they reported it as the first case of RMFSL in 2012 [2]. The authors suggested that the mutation eliminates the interaction of BRAT1 with BRCA1. BRAT1 is breast cancer 1-associated ataxia telangiectasia mutated activation-1 protein [5]. BRAT1 is a gene located on chromosome 7 that encodes a protein involved in the DNA damage pathway. Specifically, it interacts with the tumor-suppressing BRCA1 protein and binds to the ATM 1 (ataxia telangiectasia mutated 1) protein. ATM 1 plays a role in cell signaling pathways responsible for responding to DNA damage [6]. Aglipay et al. (2006) showed that BRAT1 has a role in stabilizing activated ATM protein following the DNA damage response [7].

The disease RMFSL presents as drug-resistant seizures with axial and extremity rigidity, lack of psychomotor development, and small or absent fontanelles, which were all features seen in our patient. They also can have focal jerking movements of the tongue, face, and arms. Episodic jerking could begin in utero. Newborns have microcephaly, overlapping cranial sutures, small or absent fontanelles, and depressed frontal bones. Neuroimaging is normal or reveals mild hypoplasia of the frontal lobes. Electroencephalograms show bilateral medium-high voltage spikes over temporal and central regions, frequent multifocal seizures, background slowing, and no posterior rhythm [8]. Seizures are only partially responsive to anticonvulsants and not affected by high-dose pyridoxine. They have frequent spontaneous apnea and bradycardia that uniformly culminate in cardiopulmonary arrest before the age of four months [2].

An early genetic diagnosis is essential in an infant with epileptic encephalopathy and refractory seizures to enable better management strategies [9]. A genetic diagnosis also helps in determining the prognosis and at-risk family members [10]. Clinicians should consider infantile epilepsy panel gene sequencing in all refractory neonatal or infantile seizures with features suggesting rigidity or encephalopathy. Including BRAT1 gene analysis in the infantile epilepsy gene panel is recommended.

## Conclusions

The infant in this scenario is a classic case of a rare syndrome. Lethal neonatal rigidity and multifocal seizure syndrome (RMFSL) is a severe epileptic encephalopathy that manifests with rigidity and refractory seizures. RMFSL is a recently discovered autosomal recessive disease caused by the BRAT1 gene mutations. Clinicians should consider infantile epilepsy panel gene sequencing in all refractory neonatal or infant seizures. We recommend the inclusion of BRAT1 gene analysis in the infantile epilepsy gene panel.
